# Hybrid Extended Totally Extraperitoneal Transversus Abdominis Release for Ruptured Incisional Hernia Etiologically Very Similar to Flood Syndrome: A Case Report

**DOI:** 10.70352/scrj.cr.24-00447

**Published:** 2025-01-31

**Authors:** Mariko Sambommatsu, Taketo Matsubara, Gen Shimada, Toshimi Kaido

**Affiliations:** 1 Department of Gastroenterological and General Surgery, St. Luke’s International Hospital, Tokyo, Japan; 2 Hernia Center, St. Luke’s International Hospital, Tokyo, Japan

**Keywords:** incisional hernia, Flood syndrome, liver cirrhosis, eTEP, transversus abdominis release

## Abstract

**INTRODUCTION:**

The rupture of an umbilical hernia, which is known as Flood syndrome, is a rare and life-threatening complication of liver cirrhosis. Herein, we report the successful repair of a ruptured incisional hernia accompanied by liver cirrhosis.

**CASE PRESENTATION:**

A female in her 40s with a history of alcoholic cirrhosis and ruptured acute umbilical hernia treated by primary closure 6 months earlier presented with ascites leakage from abdominal skin. She was diagnosed with a ruptured incisional hernia accompanied by massive ascites. We started preoperative management with topical corticosteroids and oral diuretics. Nine months after the first visit, hybrid herniorrhaphy, extended totally extraperitoneal transversus abdominis release (eTEP-TAR) was performed. The patient has since been well without any sign of recurrence for 2 years.

**CONCLUSIONS:**

We experienced a successful treatment of a ruptured incisional hernia accompanied by liver cirrhosis. Preoperative management and surgical strategies are important for the treatment of ruptured incisional hernia and Flood syndrome.

## Abbreviations

eTEP-TAR
: extended totally extraperitoneal transversus abdominis release
TIPS
: transjugular intrahepatic portosystemic shunt

## INTRODUCTION

Ventral hernias, especially umbilical hernias, are common complications that occur in patients with liver cirrhosis. Approximately 20% of cirrhotic patients with ascites develop umbilical hernias.^1)^ The rupture of an umbilical hernia, however, is a rare and life-threatening complication with high mortality.^2,3)^ This is also known as Flood syndrome, following the publication of a case report by Frank B. Flood in 1961.^4)^ This is a challenging condition for clinicians, as optimal management has yet to be established.

Here we report a case of ruptured incisional hernia accompanied by liver cirrhosis, which was successfully repaired by hybrid extended totally extraperitoneal transversus abdominis release (eTEP-TAR) after preoperative stabilization. Strictly, it is not a case of Flood syndrome but these management methods will provide important lessons for the treatment of ruptured incisional hernia and Flood syndrome as well.

## CASE PRESENTATION

A female in her 40s was admitted to hospital by ambulance for a sudden leakage of ascites from abdominal skin. She had a past medical history of alcoholic liver cirrhosis and ruptured umbilical hernia, which had been treated by primary closure 6 months earlier. She had noticed abdominal distension after the surgery but was not followed up for hernia. She had been taking antipsychotics for schizophrenia.

**Fig. 1:**
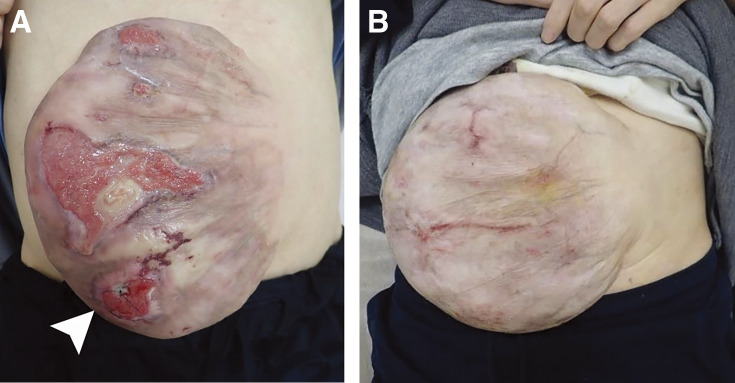
(**A**) The patient had multiple ulcerations. The one on the right lower area of the abdomen was completely ruptured and was sutured after admission (white arrowhead). (**B**) The ulcerations had completely healed.

On examination, her consciousness was clear, and she had a heart rate of 136 per min, a blood pressure of 92/72 mmHg, and a respiratory rate of 20 per min. Her body temperature was 39.4°C. She had an incisional hernia with multiple skin ulcerations, one of which was ruptured and draining at least 5 liters of serous ascites (**Fig. 1**).

**Fig. 2:**
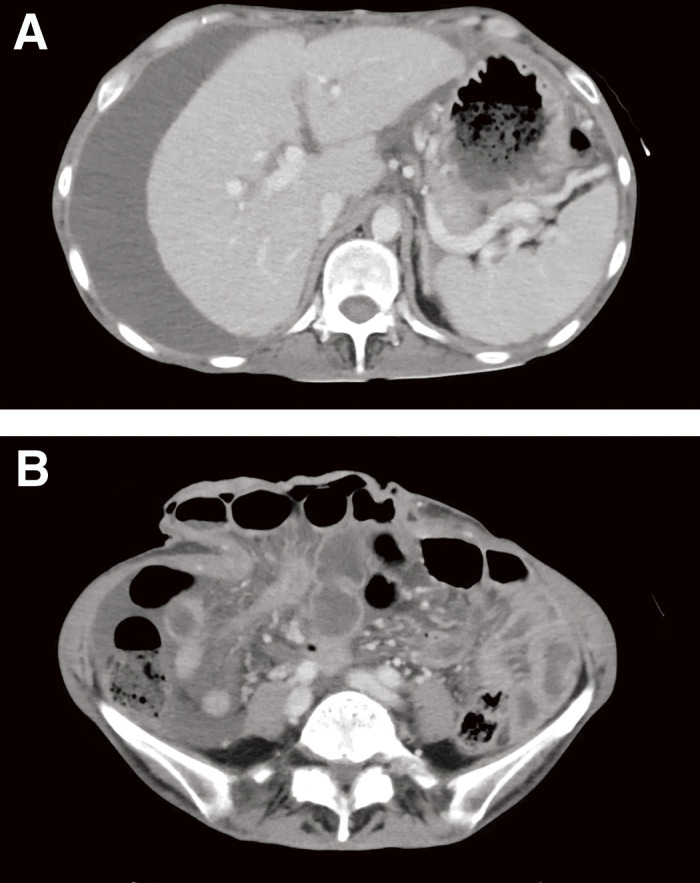
(**A**) CT scan displaying signs of liver cirrhosis, massive ascites, and hepatic atrophy. (**B**) A midline incisional hernia with a diameter of 10 cm and protrusion of the small intestine and transverse colon.

Laboratory studies revealed a hemoglobin level of 8.7 g/dL, a leukocyte count of 11000/uL and a platelet count of 225000/uL, aspartate aminotransferase (AST) level of 51 U/L, alanine transaminase (ALT) level of 20 U/L, gamma-glutamyl transferase (GGT) level of 75 U/L, and alkaline phosphatase (ALP) level of 145 U/L. Serum albumin was 2.4 g/dL and C-reactive protein (CRP) was 9.18 mg/dL. There were no signs of renal dysfunction or coagulopathy. No abnormalities were detected in the electrocardiogram and chest X-ray. A contrast-enhanced CT scan showed a midline incisional hernia with a diameter of 10 cm and protrusion of the small intestine and transverse colon. Signs of liver cirrhosis, massive ascites, hepatic atrophy, portosystemic collateral pathways, and splenomegaly were also found (**Fig. 2**). She was diagnosed with a ruptured incisional hernia accompanied by chronic liver cirrhosis with Child-Pugh score of 9 (grade B).

She was suspected to be in the condition of septic or hypovolemic shock due to massive ascites. After the initial treatment with antibiotics (tazobactam/piperacillin) and fluid resuscitation, her vital signs became stable. As the ascites leakage decreased after we sutured the ruptured skin ulceration, she was admitted to the hospital. We continued the antibiotics for 6 days, and the blood culture taken on the day of admission was negative. At the same time, we started nonoperative management with topical corticosteroids and oral diuretics (tolvaptan 3.75 mg/day, furosemide 20 mg/day, and spironolactone 25 mg/day). As the skin ulcerations showed improvement, she was discharged 6 days after hospitalization. She continued outpatient visits for 9 months, and skin ulcerations had completely healed (**Fig. 1**). As she quit drinking alcohol and continued oral diuretics, her liver cirrhosis improved markedly with decreased ascites (Child-Pugh grade B to A).

**Fig. 3:**
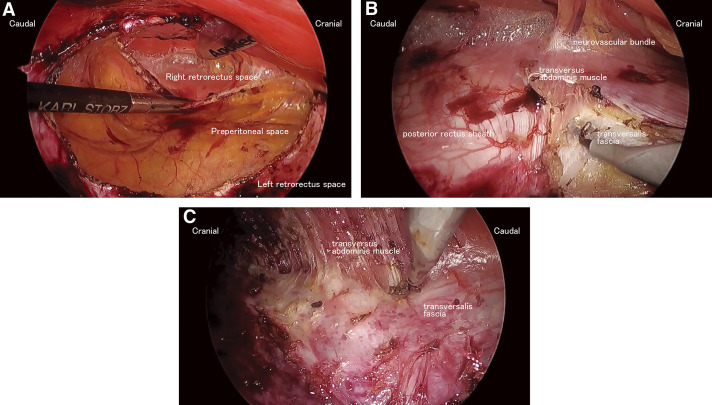
(**A**) Crossover was performed to enter the right retrorectus space. (**B**) Right TAR was performed by the top-down approach. (**C**) Left TAR was performed by the bottom-up approach. TAR, transversus abdominis release

A hybrid hernioplasty was then planned. We started retrorectus dissection using the e-TEP technique. A crossover was performed to enter the contralateral retrorectus space, and then both retrorectus spaces were dissected. A bilateral TAR was performed, and the posterior layer was closed with a 2-0 barbed suture. Then, an incision was made over the defect to remove atrophic skin. The hernia orifice was closed, and 34 × 26 cm of polypropylene mesh was placed. Drains were replaced in the subcutaneous space and retrorectus space (**Fig. 3**).

**Fig. 4:**
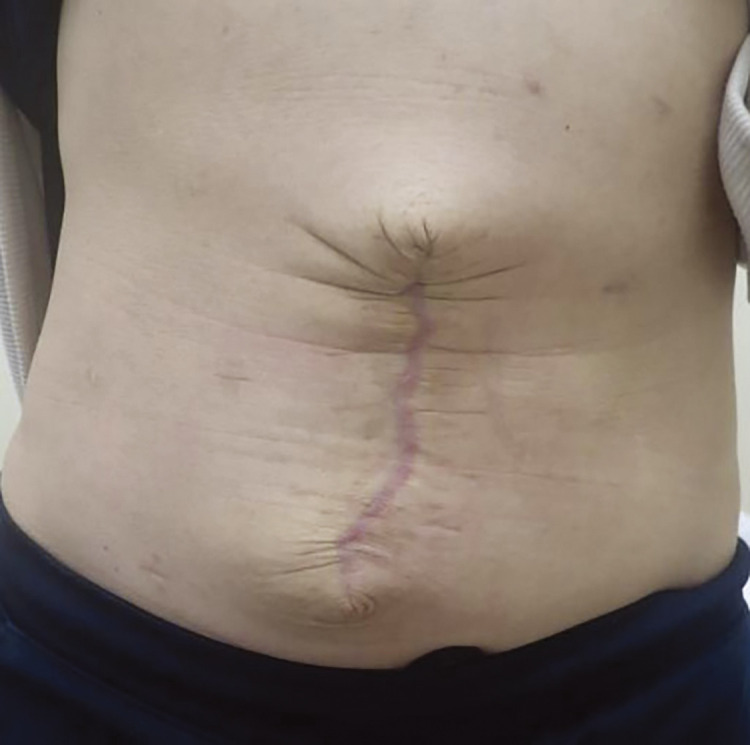
The patient had no wound complication postoperatively.

The postoperative course was uneventful. The patient was administered albumin infusion 3 days before and after surgery. The subcutaneous drain and the retrorectus drain were removed on postoperative days 4 and 6, respectively, and the patient was discharged on postoperative day 7. Two years after surgery, she is well with no complications such as wound infection, hernia recurrence, or ascites leakage (**Fig. 4**).

## DISCUSSION

Original Flood syndrome refers to a sudden drainage of ascites accompanied by spontaneous rupture of an umbilical hernia.^4)^ Strictly, this is not a case of Flood syndrome, but we consider this case to have almost the same etiology as Flood syndrome and be helpful in discussing the treatment of ruptured incisional hernia and flood syndrome.

Possible complications of the ruptured ventral hernia are the incarceration of the intestines, hemodynamic instability, electrolyte abnormalities, and infection, including cellulitis and peritonitis.^3–5)^ Mortality rates following urgent surgical repair of a hernia range from 6 to 20%, but they increase to around 70% with only supportive care.^3)^ Surgery appears to be a life-saving intervention; however, it is possible that those who were not operated on were already sicker at baseline. Additionally, there is no reliable data on the optimal duration of conservative treatment.^6)^ Therefore, the management of Flood syndrome is challenging for clinicians, and it is the subject of much debate as clear recommendations are lacking.

Regarding preoperative management, stabilization and local wound care are key points. The optimal control of ascites is one of the main goals, to prevent wound infection and hernia recurrence.^7)^ The strategies for controlling ascites include sodium intake restriction, diuretic therapy, and, if needed, transjugular intrahepatic portosystemic shunt (TIPS) in refractory cases.^8)^ Local wound care is also important to prevent infection and excessive ascites loss. Macerations at the hernia site may be closed with Z sutures with an occlusive dressing or an ostomy bag to collect the remaining ascites.^6)^

There is no consensus regarding surgical methods for Flood syndrome. Most authors suggest a primary closure with non-absorbable sutures with concerns for infectious complications.^6,7)^ A single randomized study of 80 patients, however, demonstrated that the use of mesh in cirrhotic patients with complicated umbilical hernia was related to a lower rate of hernia recurrence.^9)^ This case was a recurrence of ventral hernia after the primary closure for umbilical hernia rupture, so elective herniorrhaphy with mesh was favorable.

Hybrid eTEP-TAR combines minimally invasive retromuscular and preperitoneal dissection with open techniques for fascial closure, mesh placements, and atrophic skin excision. Addo et al. revealed a decrease in the length of hospital stay and a decrease in overall postoperative complications in patients receiving hybrid ventral hernia repair compared to open surgery.^10)^ Additionally, Ahonen-Siirtola et al. reported the benefit of adhesiolysis through a minimally invasive open technique.^11)^ If the clinical situation allows elective herniorrhaphy after preoperative optimization, hybrid approaches can be an option, even in cases of ruptured incisional hernia.

## CONCLUSION

We experienced a case of ruptured incisional hernia following umbilical hernia repair in a patient with liver cirrhosis. Preoperative management and surgical strategies are important for the treatment of ruptured incisional hernia and Flood syndrome.

## ACKNOWLEDGMENTS

None.

## DECLARATIONS

### Funding

We received no financial support for the research, authorship, and/or publication of this article.

### Authors’ contributions

MS and TM carried out the experiment.

MS wrote the manuscript with support from TM and GS.

TK helped supervise the project.

### Availability of data and materials

Not applicable.

### Ethics approval and consent to participate

Not applicable.

### Consent for publication

Informed consent was obtained from the patient for publication.

### Competing interests

There are no potential conflicts of interest with respect to the research, authorship, and publication of this article.
